# Radiofrequency ablation for anti-gastroesophageal reflux under direct vision via nasal endoscopy

**DOI:** 10.1055/a-2619-1089

**Published:** 2025-07-09

**Authors:** Wen Xu, Zhengxia Lei, Qing Cheng, Laihe Li, Ying Zhu

**Affiliations:** 1 559569 Department of Gastroenterology, Shenzhen Hospital of Southern Medical University, Shenzhen, China

A 62-year-old woman presented with a decade-long history of recurrent acid reflux and heartburn. Upper gastrointestinal contrast radiography and gastroscopy confirmed gastroesophageal reflux disease (GERD), classified as LA-A (Los Angeles classification), and revealed an esophageal hiatal hernia ( Fig. 1 ). Esophageal manometry showed a lower esophageal sphincter (LES) resting pressure of 9mmHg (normal range: 10–36mmHg), and dynamic reflux monitoring indicated an acid exposure time of 13.3% with a DeMeester score of 21.5.

**Fig. 1:**
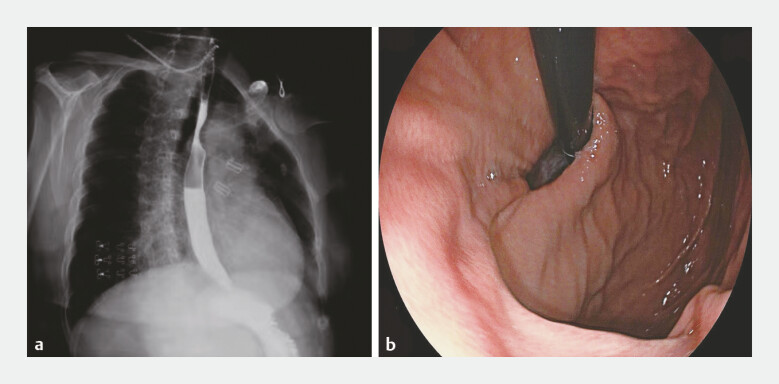
**a** Preoperative upper gastrointestinal contrast radiography revealed gastroesophageal reflux. **b** Preoperative gastroscopy indicated esophageal hiatal hernia.

To alleviate symptoms, radiofrequency ablation was considered. During the procedure, a radiofrequency catheter was inserted via gastroscopy, and its position was confirmed using nasal endoscopy ( Fig. 2 **a, b** ), ensuring precise placement by direct visualization. Once positioned, the nasal gastroscope was withdrawn, and the catheter’s balloon was inflated, deploying four needles into the muscularis propria layer. Energy was delivered to the cardia and the area spanning 1.5cm above to 1.5cm below the dentate line, with treatments spaced 0.5cm apart. A total of 80 lesions were placed across nine levels. Post-procedure gastroscopy confirmed lesion placement ( Fig. 2 **c, d** , Video 1 ). The patient tolerated a liquid diet 24 hours after the operation without complications. Follow-up gastroscopy 6 months later showed a tightened cardia, and the patient reported significant improvement in reflux symptoms ( Fig. 2 **e** ).

**Fig. 2:**
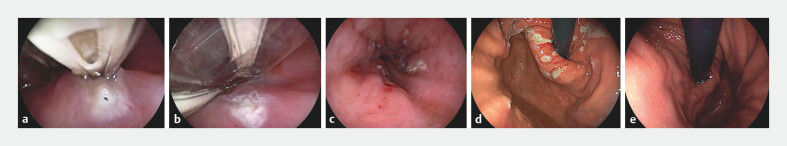
**a** The catheter was placed via gastroscopy. **b** The location of the catheter was determined by nasal endoscopy. **c, d** Postoperatively, the radiofrequency lesions were observed by gastroscopy: **c** lower esophagus, **d** cardia. **e** Re-examination by gastroscopy conducted 6 months after operation showed the cardia had tightened.

Radiofrequency ablation procedure under direct vision via nasal endoscopy, with re-examination via gastroscopy after the procedure.Video 1

Radiofrequency ablation offers a promising antireflux treatment for refractory GERD 1 , working by inhibiting LES relaxation, increasing LES pressure, and reducing esophageal hypersensitivity 2 3 . This case highlights the use of nasal endoscopy for precise catheter positioning, overcoming limitations of traditional methods that rely on gastroscopy measurements, thus enhancing treatment accuracy and outcomes.

Endoscopy_UCTN_Code_TTT_1AO_2AJ

## Notes

Endoscopy E-Videos https://eref.thieme.de/e-videos
*E-Videos* is an open access online section of the journal *Endoscopy* , reporting on interesting cases and new techniques in gastroenterological endoscopy. All papers include a high-quality video and are published with a Creative Commons CC-BY license. Endoscopy E-Videos qualify for HINARI discounts and waivers and eligibility is automatically checked during the submission process. We grant 100% waivers to articles whose corresponding authors are based in Group A countries and 50% waivers to those who are based in Group B countries as classified by Research4Life (see: https://www.research4life.org/access/eligibility/ ). This section has its own submission website at https://mc.manuscriptcentral.com/e-videos .
